# Hypermobility prevalence, measurements, and outcomes in childhood, adolescence, and emerging adulthood: a systematic review

**DOI:** 10.1007/s00296-023-05338-x

**Published:** 2023-05-06

**Authors:** Liron Blajwajs, Joanne Williams, Wendy Timmons, John Sproule

**Affiliations:** 1 Institute of Sport, Physical Education and Health Sciences, The University of Edinburgh, Edinburgh, UK; 2 Department of Clinical and Health Psychology, School of Health in Social Science, The University of Edinburgh, Edinburgh, UK

**Keywords:** Hypermobility, Generalised Joint Hypermobility

## Abstract

General Joint Hypermobility (GJH) is a common condition found in 2–57% of the population. Of those with GJH, 10% suffer from accompanying physical and/or psychological symptoms. While the understanding of GJH in the general population is unfolding, its implication in a cohort of children, adolescents and young adults are not yet understood. This systematic review explored GJH’s prevalence, tools to measure it, its physical and psychosocial symptoms, with a special interest in aesthetic sports. The CINHAL, MEDLINE, PsycINFO, SPORTDiscus and Scopus databases were searched for relevant studies. Inclusion criteria were (1) Age range of 5–24; (2) Participants had GJH; (3) A measurement for GJH; (4) Studies written in English language. Study screening for title, abstract and full text (when needed) and quality assessment were performed by two independent individuals. 107 studies were included in this review and were thematically grouped into six clusters expressing different foci: (1) GJH’s Core Characteristics; (2) Orthopedic; (3) Physical Other; (4) Psychosocial; (5) Treatment and (6) Aesthetic Sports. The review revealed a growing interest in GJH in this cohort in the last decade, especially regarding non-musculoskeletal physical implications and psychosocial aspects. Prevalence varied between different ethnic groups and as a parameter of age, gender and measurement. The most widespread tool to measure GJH was the Beighton scale, with a cut-off varying between 4 and 7. Children show fewer, but similar GJH implication to those in the general population, however, more research on the topic is warranted, especially regarding psychosocial aspects and treatment.

## Introduction

This systematic review of empirical research focuses on the health and psychological symptoms related to hypermobility in children, adolescents, and young adults. The term hypermobility is defined as increased Range of Motion (ROM) in the joints, considering age, gender, and ethnicity [1]. While such ROM can be acquired, this study’s interest lies in heritable hypermobility, which is a Heritable Disorder of Connective Tissue (HDCT). HDCTs are a group of 200 genetic disorders affecting connective tissue matrix protein, leading to structural, functional, and biomechanical abnormalities that can manifest in tissue fragility and malfunction and can be difficult to diagnose [1, 2]. The most common of the HDCTs, are Hypermobility Spectrum Disorders (HSD) and in particular, Joint Hypermobility (JH). JH is a Heritable Spectrum of four Disorders (HSD) [3], Localized JH, Generalized JH, Peripheral JH, and Historical JH. Within HSD, Generalized JH (GJH) is the most prominent and common and has clear criteria for clinical observation; accordingly, GJH was the focus of this review. GJH presents in a general display (involving the whole body) occurring in 2–57% of the population [4]. Of those with GJH, 10% suffer from physical and/or psychological symptoms with 0.5–2% estimated prevalence [5]. It can present as symptomatic or asymptomatic and is widely measured by the Beighton Scale (BS) [6]. The BS is an observational tool considered the most reliable and valid for measuring JH in both pediatric and adult populations, to date. However, it was recently advised to use it carefully, especially in children, where its validity is unclear [7].

As the research field is still emerging and the classifications of JH have changed with new guidelines coming out in 2017, the present study, therefore, aimed to examine GJH’s operationalization and trends in research over the years. Another caveat rising from the literature, is the scope of GJH in a younger population of children, adolescents, and young adults, who present different symptoms, for the most part, less debilitating than adults [8]. Consequently, this study explored GJH’s physical implications in this particular population. It identified different tools used to screen for GJH and determined its prevalence in children and adolescents. Moreover, it aimed to identify psychosocial implications of GJH in youth, including quality of life and mental health disorders with a view to highlight areas that require further research regarding GJH’s psychosocial aspects in this cohort. The review also focused on aesthetic sports and dance contexts, as it has been suggested that there are special considerations for people with hypermobility engaging in these activities, such as the need for different measurements as well as advantages and disadvantages it can pose [9].

## Methods

### Search strategy

**Fig. 1:**
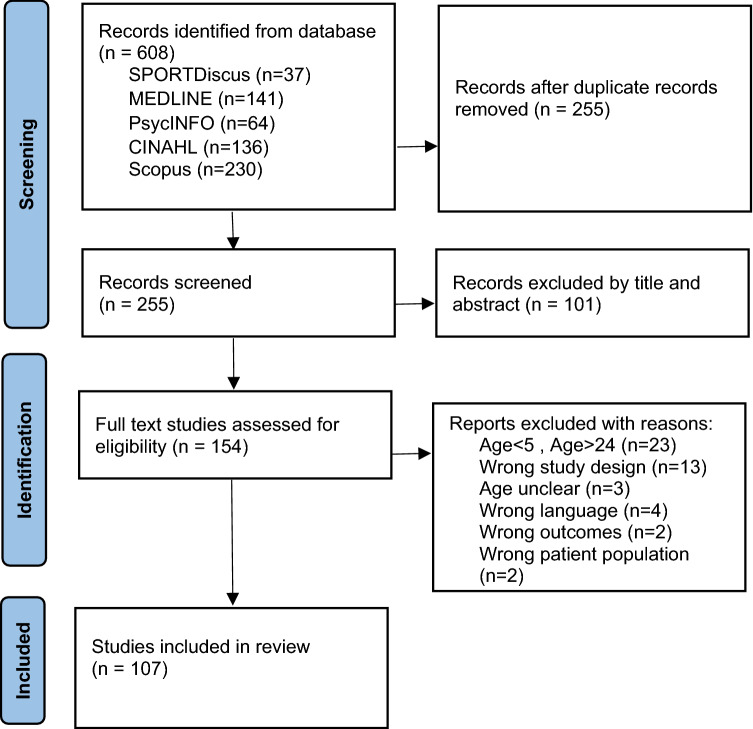
PRISMA flow diagram

The systematic review was performed by electronic searches on April 11th, 2023 in CINAHL (*n* = 136), SPORTDiscus (*n* = 37), PsycINFO (*n* = 64), MEDLINE (*n* = 141), and Scopus (*n* = 230). Previous hypermobility systematic reviews were screened and the searches were trialed and refined. In each of the five databases, the following search terms were used in the title field (hypermobility or “joint hypermobility” or “general joint hypermobility” or “joint hypermobility syndrome” or “benign joint hypermobility syndrome” or hyperlaxity or “joint laxity” or “joint flexibility” or “hypermobile spectrum disorders” or “connective tissue disorders” or “collagen abnormalities”) AND (child or children or teen* or teenager* or student* or undergraduate* or youth or “young people” or “young adult” or pediatric). See Fig. 1 for selection process.

### Study selection

This systematic review followed the PRISMA statement guidelines [10], and was guided by the PICOS method for the search strategy: Participants (children, adolescents, and young adults with and without GJH, between the age of 5 and 24), Intervention (presence of GJH), Comparison (healthy controls), Outcomes (tools for measuring GJH, prevalence of GJH, physical and psychological implications of GJH), and Study design (any study where data was obtained). The review protocol was submitted in the International Prospective Register of Systematic Reviews (https://www.crd.york.ac.uk/PROSPERO/display_record.php?RecordID=259272).

### Selection criteria

Included articles had to meet the following criteria: (1) participants were children, adolescents or young adults aged 5–24 years; (2) participants had GJH; (3) there was a clinical assessment method to classify GJH; and (4) the study was reported in English. Studies were excluded if: (1) participants were younger than 5 years old or older than 24; (2) participants did not have GJH; (3) the studies had no clear measurement for GJH; or (4) the studies were commentaries or reviews.

### Data extraction

Two of the authors independently screened titles and abstracts for relevant studies. Any conflicts were resolved in a discussion with the full paper retrieved for further assessment when necessary, and consensus was achieved. Handling of data was done through Covidence, for easy screening and data extraction.

### Main outcome variables

This study was interested in childhood GJH including prevalence, tools to measure it, and its physical as well as psychosocial symptoms.

### Risk of bias assessment

Quality assessment was rated independently by two authors using the Effective Public Health Practice Project (EPHPP) quality assessment tool for quantitative studies [11]. Any conflicts were resolved by discussion. EPHPP addresses selection bias, study design, confounders, blinding, data collection, and withdrawals. A study’s global rating can range between: ‘strong’ = no weak subscale ratings; ‘moderate’ = one weak subscale rating; and ‘weak’ = two or more weak subscale ratings.

### Analysis

This review used a narrative synthesis.

## Results

The electronic database search identified 608 articles. After removal of duplicates, 255 studies were screened for title, abstract, and full text, when needed. One hundred and seven studies met inclusion criteria and were included in the current review. Fifty-four studies were rated “weak”, fifty-one were rated “moderate”, and two were rated “strong”. This was mostly due to weaker study designs, with 78 cross-sectional studies (see Table 1).

**Table 1:** Quality assessment

Reference	Selection bias	Study design	Confounders	Blinding	Data collection methods	Withdrawal and dropouts	Total
Abujam, 2014 [12]	Moderate	Weak	Moderate	Moderate	Moderate	NA	Moderate
Akalan, 2018 [13]	Moderate	Weak	Weak	Moderate	Weak	NA	Weak
Akkaya, 2022 [14]	Moderate	Weak	Moderate	Weak	Strong	NA	Weak
Akkaya, 2023 [15]	Moderate	Weak	Moderate	Moderate	Weak	NA	Weak
BarÇAk, 2015 [16]	Moderate	Weak	Weak	Moderate	Weak	NA	Weak
Barron, 2002 [17]	Moderate	Weak	Strong	Moderate	Moderate	NA	Moderate
Bayramoğlu, 2020 [18]	Moderate	Weak	Moderate	Moderate	Weak	NA	Weak
Bettini, 2018 [19]	Moderate	Weak	NA	NA	Moderate	NA	Moderate
Bettini, 2016 [20]	Moderate	Weak	Weak	Moderate	Moderate	NA	Weak
Bieniak, 2022 [21]	Moderate	Weak	NA	NA	Strong	NA	Moderate
Bieniak, 2022b [22]	Moderate	Weak	NA	NA	Strong	NA	Moderate
Birt, 2014 [23]	Moderate	Strong	Strong	Moderate	Weak	Weak	Weak
Boris, 2021 [24]	Moderate	Weak	Strong	Moderate	Weak	NA	Weak
Bozkurt, 2019 [25]	Moderate	Weak	Weak	Moderate	Weak	NA	Weak
Bulbena-Cabre, 2019 [26]	Moderate	Weak	Strong	Strong	Moderate	NA	Moderate
Can, 2022 [27]	Modearte	Weak	Moderate	Moderate	Strong	Weak	Weak
Carr, 1993 [28]	Moderate	Weak	Weak	Moderate	Weak	NA	Weak
Chelimsky, 2016 [29]	Weak	Weak	Weak	Moderate	Weak	NA	Weak
Clinch, 2011[30]	Moderate	Weak	Weak	Moderate	Weak	NA	Weak
Czaprowski, 2015 [31]	Moderate	Weak	Moderate	Moderate	Moderate	NA	Moderate
Czaprowski, 2013 [32]	Moderate	Weak	Strong	Moderate	Weak	NA	Weak
Czaprowski, 2017 [33]	Moderate	Moderate	Moderate	Weak	Moderate	NA	Moderate
Czaprowski, 2012 [34]	Moderate	Weak	Weak	Weak	Weak	NA	Weak
Czaprowski, 2021 [35]	Moderate	Weak	Moderate	Moderate	Moderate	NA	Moderate
Davidovitch, 1994 [36]	Moderate	Weak	Moderate	Moderate	Moderate	NA	Moderate
de Boer, 2015 [37]	Moderate	Moderate	Moderate	Moderate	Strong	Weak	Moderate
de Kort, 2003 [38]	Weak	Weak	Weak	Moderate	Moderate	NA	Weak
Demir, 2021 [39]	Moderate	Weak	Moderate	Moderate	Weak	NA	Weak
Dobies-Krześniak, 2022 [40]	Moderate	Weak	Moderate	Moderate	Strong	NA	Moderate
El Garf, 1998 [41]	Moderate	Weak	NA	NA	Weak	NA	Weak
Engelbert, 2003 [42]	Moderate	Weak	Moderate	Moderate	Moderate	NA	Moderate
Engelbert, 2006 [43]	Moderate	Weak	Weak	Moderate	Moderate	NA	Weak
Evrendilek, 2019 [44]	Strong	Weak	Strong	Strong	Strong	NA	Moderate
Ezpeleta, 2018 [45]	Weak	Moderate	NA	NA	Moderate	Moderate	Moderate
Fairbank, 1984 [46]	Moderate	Weak	NA	NA	Weak	NA	Weak
Falkerslev, 2013 [47]	Moderate	Weak	Strong	Moderate	Weak	NA	Weak
Fato8ye, 2009 [48]	Moderate	Weak	Weak	Moderate	Weak	NA	Weak
Fatoye, 2011 [49]	Moderate	Weak	Weak	Moderate	Strong	NA	Weak
Fatoye, 2012 [50]	Moderate	Weak	Weak	Moderate	Strong	NA	Weak
Fernanzed-Bermejo, 1993 [51]	Moderate	Weak	Weak	Moderate	Weak	NA	Weak
Forleo, 1993 [52]	Moderate	Weak	NA	NA	Weak	NA	Weak
Frohlich, 2012 [53]	Weak	Moderate	NA	NA	Strong	NA	Moderate
Gocentas, 2016 [54]	Moderate	Weak	NA	NA	Weak	NA	Weak
Graf, 2019 [55]	Moderate	Weak	NA	NA	Moderate	NA	Moderate
Gyldenkerne, 2007 [56]	Strong	Moderate	Moderate	Moderate	Weak	NA	Moderate
Hanewinkel-Van Kleef, 2009 [57]	Moderate	Weak	NA	NA	Strong	NA	Moderate
Hickey, 2016 [58]	Weak	Weak	Weak	Moderate	Strong	NA	Weak
Hornsby, 2022 [59]	Weak	Weak	NA	Weak	Strong	NA	Weak
Ilgunas, 2020 [60]	Weak	Weak	NA	NA	Weak	NA	Weak
Jansson, 2004 [61]	Moderate	Moderate	NA	NA	Weak	NA	Moderate
Jensen, 2013 [62]	Moderate	Weak	Strong	Moderate	Weak	NA	Weak
Junge, 2013 [63]	Moderate	Weak	NA	Moderate	Strong	NA	Moderate
Junge, 2015 [64]	Strong	Moderate	Moderate	Moderate	Strong	Weak	Moderate
Juul-Kristensen, 2012 [65]	Moderate	Weak	Strong	Moderate	Strong	NA	Moderate
Juul-Kristensen, 2009 [66]	Moderate	Weak	Moderate	Moderate	Strong	NA	Moderate
Kajbafzadeh, 2014 [67]	Moderate	Weak	Strong	Moderate	Weak	NA	Weak
Karademir, 2022 [68]	Weak	Weak	NA	NA	Weak	NA	Weak
Kendel, 2019 [69]	Weak	Weak	NA	NA	Moderate	NA	Weak
Kendel, 2022 [70]	Strong	Weak	NA	NA	Strong	NA	Moderate
Kindgren, 2021 [71]	Moderate	Weak	NA	NA	Weak	NA	Weak
Kubasadgoudar, 2012 [72]	Moderate	Weak	NA	NA	Moderate	NA	Moderate
Leonardis, 2021 [73]	Weak	Weak	NA	NA	Weak	NA	Weak
McDermott, 2018 [74]	Moderate	Weak	NA	NA	Strong	NA	Moderate
Moore, 2019 [75]	Weak	Weak	Weak	Moderate	Moderate	NA	Weak
Morris, 2017 [76]	Strong	Weak	NA	NA	Strong	NA	Moderate
Mosulishvili, 2013 [77]	Moderate	Strong	Moderate	Weak	Weak	Weak	Weak
Mu, 2019 [78]	Weak	Weak	NA	NA	Weak	NA	Weak
Nash, 2017 [79]	Weak	Weak	NA	NA	Weak	NA	Weak
Nicholson, 2017 [80]	Moderate	Weak	Moderate	Moderate	Strong	NA	Moderate
Nikolajsen, 2021 [81]	Weak	Weak	Moderate	Weak	Weak	NA	Weak
Nikolajsen, 2013 [82]	Moderate	Weak	Moderate	Moderate	Strong	NA	Moderate
Nilsson, 1993 [83]	Moderate	Weak	Moderate	Moderate	Weak	NA	Weak
Öhman, 2014 [84]	Moderate	Weak	NA	Moderate	Strong	NA	Moderate
Önerge, 2018 [85]	Moderate	Weak	Weak	Moderate	Weak	NA	Weak
Ortiz-Rivera, 2022 [86]	Moderate	Weak	Strong	Moderate	Weak	NA	Weak
Pacey, 2014[87]	Moderate	Weak	NA	NA	Strong	NA	Moderate
Pacey, 2013 [88]	Moderate	Strong	Strong	Strong	Strong	Strong	Strong
Pacey, 2015 [89]	Moderate	Weak	NA	NA	Strong	NA	Moderate
Pacey, 2015b [90]	Moderate	Weak	Strong	Moderate	Strong	NA	Moderate
Parvaneh, 2020 [91]	Moderate	Weak	Strong	Moderate	Moderate	NA	Moderate
Pitetti, 2015 [92]	Strong	Weak	NA	NA	Moderate	NA	Moderate
Rejeb, 2019 [93]	Moderate	Weak	Strong	Moderate	Moderate	NA	Moderate
Remvig, 2011 [94]	Strong	Weak	Moderate	Moderate	Strong	NA	Moderate
Revivo, 2019 [95]	Moderate	Moderate	Moderate	Moderate	Strong	Weak	Moderate
Şahin, 2023 [96]	Moderate	Weak	Moderate	Moderate	Weak	NA	Weak
Salem, 2010 [97]	Weak	Moderate	NA	NA	Weak	NA	Weak
Sanjay, 2013 [98]	Moderate	Weak	NA	Moderate	Weak	NA	Weak
Santos, 1981 [99]	Moderate	Weak	Strong	Moderate	Weak	NA	Weak
Scheper, 2017 [100]	Moderate	Moderate	Moderate	Moderate	Strong	Weak	Moderate
Schmidt, 2017 [101]	Moderate	Weak	Moderate	Moderate	Strong	NA	Moderate
Schubert-Hjalmarsson, 2012 [102]	Weak	Weak	Weak	Moderate	Strong	NA	Weak
Seçkin, 2005 [103]	Strong	Weak	NA	NA	Weak	NA	Weak
Shulman, 2020 [104]	Moderate	Weak	Moderate	Moderate	Strong	NA	Moderate
Sirajudeen, 2020 [105]	Strong	Weak	NA	NA	Strong	NA	Moderate
Smits-Engelsman, 2014 [106]	Moderate	Weak	NA	NA	Strong	NA	Moderate
Sohrbeck-Nøhr, 2014 [107]	Strong	Moderate	Strong	Moderate	Strong	Strong	Strong
Subramanyam, 1996 [108]	Moderate	Weak	NA	NA	Weak	NA	Weak
Tobias, 2013 [109]	Strong	Moderate	Moderate	NA	Weak	Weak	Weak
Tokhmafshan, 2020 [110]	Strong	Weak	NA	NA	Moderate	NA	Moderate
Tran, 2020 [111]	Moderate	Weak	Moderate	Moderate	Strong	NA	Moderate
Velasco-Benitez, 2020 [112]	Moderate	Weak	Strong	Moderate	Moderate	NA	Moderate
Velasco-Benitez, 2021 [113]	Modearte	Weak	Strong	Moderate	Weak	NA	Weak
Woolston, 2012 [114]	Moderate	Weak	Strong	Moderate	Weak	NA	Weak
Wright, 2020 [115]	Moderate	Weak	Strong	Moderate	Strong	NA	Moderate
Yazgan, 2008 [116]	Strong	Weak	Moderate	Moderate	Moderate	NA	Moderate

The papers in this review were thematically grouped into six clusters expressing different foci: (1) GJH core characteristics; (2) orthopedic; (3) physical other; (4) psychosocial; (5) treatment, and (6) aesthetic sports (see Table 2). Twelve studies fit into more than one category (e.g., a prevalence study examining orthopedic problems) and were included in both.

**Table 2:** Thematic clusters

Name of cluster	Description	Number of studies included	Range of years
GJH’s core characteristics	Includes studies focusing on the core characteristics of GJH: (a) prevalence in different population with different ethnicities (Indian, Brazilian, Swedish, Egyptian, and more), and different ages (children and adolescents); (b) tool to measure GJH; (c) a general overview on this condition looking at its signs and symptoms. Such studies have been published since the 1980’s with a consistent focus throughout the years	33	1981–2022
Orthopedic implications	Includes papers exploring the different joints affected by GJH, such as the spine, hips, knees, shoulders, and jaw. Studies within the cluster explored motor competence and development, muscle strengths, gait patterns, biomechanics of the joints and musculoskeletal pain. This cluster is the largest one with a consistent interest starting in the mid 1980’s till this day	45	1984–2023
Other physical implications	Includes studies looking at physical implications that are not musculoskeletal, either focusing on large systems of the body such as the cardiovascular and autonomic system, the gastrointestinal system gynecological problems, incontinence problems, or at local symptoms, such as eye and dental problems. Most of the studies in this cluster were published in the last couple of decades	21	2003–2023
Psychosocial implications	Includes research about the psychosocial implications of GJH, such as mental disorders (especially anxiety), well-being, and learning difficulties. Papers exploring well-being emerged in 2011, while studies on anxiety disorders were first published in 2018. A single study on learning difficulties stands out, as it was published in 1994	13	1994–2022
Treatments	Includes studies looking at interventions to help the debilitating effects of GJH, such as physiotherapy, emotional therapy, and orthopedic aids. Such studies have emerged in the last 15 years	10	2007–2022
Aesthetic sports context	Includes papers exploring how GJH affects the participants in aesthetic sports and dance, regarding their performance, physical attributes, and well-being	3	1993–2017

### Synthesis of findings by theme

#### GJH characteristics

First, this cluster includes studies exploring prevalence of GJH in specific populations, such as children of various ages from ten different countries (see Table 3(1:A)).

**Table 3:** Results

(1) Cluster 1: core characteristics of GJH					
A: Prevalence					
Country	Age	Total GJH	Boys	Girls	References
Brazil	6–17	2.3%	–	–	Santos, 1981 [99]
	5–17	36.0%	33.7%	38.4%	Forleo, 1993 [52]
Denmark	12–13	9.4%	3.3%	16.6%	Gyldenkerne, 2007 [56]
	10	35.6%	33.3%	37.7%	Remvig, 2011 [94]
Egypt	6–15	16.1%	14.0%	18.0%	El-Garf, 1998 [41]
India	6–15	17.2%	19.4%	15.0%	Subramanyam, 1996 [108]
	6–12	34.3%	33.7%	35.1%	Kubasadgoudar, 2012 [72]
Lithuania	10–18	19.2%	–	–	Gocentas, 2016 [54]
Pakistan	8–17	33.5%	29%	30.8%	Butt, 2014 [117]
Saudi Arabia	8–14	16.8%	13.4%	15.2%	Sirajudeen, 2020 [105]
	6–12	13.5%	30.87%	25.6%	Al-Shenqiti, 2022 [118]
Sweden	9,12,15	34.9%	24.8%	45.6%	Jansson, 2004 [61]
Turkey	13–19	11.7%	7.2%	16.2%	Seçkin, 2005 [103]
	11–18	9.1%	6.1%	12.4%	Barçak, 2015 [16]
	10–15	18.4%	16.7%	20.5%	Bozkurt, 2019 [25]
The UK	14	19.2%	10.6%	27.5%	Clinch, 2011 [30]

B: Underlying conditions, injury and pain					
Cohort*	Age	Measured	Patients*	Controls*	References
Scoliosis	10–15	GJH	6.5%	4.9%	Bozkurt, 2019 [25]
	7–18	GJH	51%	41%	Dobies-Krześniak, 2022 [40]
GI problems	7–12	GJH	35.0%	36.0%	Shulman, 2020 [104]
Vesicoureteric Reflux+	≥ 6	GJH	66.48%	–	Tokhmafshan, 2020 [110]
POTS	14–17	Symptomatic GJH	61.7%	38.3%	Boris, 2021 [24]
Anxiety	8–15	GJH	52.0%	16.0%	Parvaneh, 2020 [91]
GJH	12–13	Injury Pain	75.0% 88.0%	45.0% 69.0%	Gyldenkerne, 2007 [56]
GJH	10–18	POTS & OH	2.3%	1.8%	Velasco-Benitez, 2021 [113]
*Patients with the condition in the cohort column and control without +No control group					

C: Signs and symptoms			
Number of clusters	Age	Description	References
5	6–16	1. Joint affected 2. Athletic 3. Systematic 4. Soft-tissue affected 5. High BMI	Pacey, 2015 [89]
3	6–18	1. Mild 2. Moderate 3. Severe	Scheper, 2017 [100]

(2) Cluster 2: Orthopedic implications of GJH			
A: Involved joints			
Joint of interest	Condition	Age	References
Spine and scoliosis	Back pain	13–17	Fairbank, 1984 [46]
	Idiopathic scoliosis	13–19	Fernandez-Bermejo, 1993 [51]
	Idiopathic scoliosis	9–18	Czaprowski, 2012 [34]
	Sagittal spine profiles	10–13	Czaprowski, 2013 [32]
	Sagittal spine curvature	10–14	Czaprowski, 2017 [33]
	Sagittal body alignment in a sitting position	8–14	Czaprowski, 2021 [35]
	Back mobility	5–17	Woolston, 2012 [114]
	Idiopathic scoliosis	10–15	Bozkurt, 2019 [25]
Knee	Knee ROM and gait	8–15	Fatoye, 2011 [49]
	Knee function	10	Juul-Kristensen, 2012; Jensen, 2013 [62, 65]
	Knee injuries	9–14	Junge, 2015 [64]
	Knee muscle activation and kinematics	14–15	Nikolajsen, 2021 [81]
Hip	Hip rotation	7–8	Carr, 1993 [28]
	Femoral anteversion	Av. 8.5	Akalan, 2018; Önerge, 2018 [13, 85]
Jaw	Temporomandibular dysfunction and pain	6–16	Demir, 2021 [39]
	Temporomandibular hypermobility	10–18	Graf, 2019 [55]
	Jaw disorders	18–22	Ilgunas, 2020 [60]
	Jaw mobility	5–17	Woolston, 2012 [114]
Shoulder	Shoulder sensorimotor activity and neuromuscular control	13–17	Frydendal, 2018 [119]
	Three-dimensional shoulder motions	Av. 14	Leonardis, 2021 [73]

B: Orthopedic implications of GJH				
Subject of interest	Measurement	Age	Association with GJH	References
Motor competence	1.Muscle strength 2. Exercise capacity 3. Motor performance	5–12	1. Moderate decrease 2. Significant impairment 3. Delay	Hanewinkel-van Kleef, 2009 [57]
	1. Motor performance 2. Muscle strength 3. Neuromuscular performance	6–12	1. Not associated 2. Not associated 3. Not associated	Wright, 2020 [115]
	1. Motor performance 2. Pain 3.Injury 4. Physical activity	10	1. Inconclusive 2. Not associated 3. GJH > control 4. Not associated	Remvig, 2011 [94]
	1. Motor performance 2. Pain and injury 3. Physical activity	8	1. GJH > control 2. Not associated 3. Not associated	Juul-Kristensen, 2009 [66]
	1. Body awareness 2. Muscle strength 3. Physical fitness assessment 4. Pain	18–24	1. Not associated 2. GJH < control 3. GJH < control 4. GJH > control	Akkaya, 2022 [14]
DCD	DCD	6–12	Not associated	Moore, 2019 [75]
	1. Motor developmental rate 2. Motor performance	5.5	1. GJH > control (moderately) 2. GJH > control (moderately)	De Boer, 2015 [37]
Gait	Gait patterns with/without GJH	9–11	Differences with/without GJH	Nikolajsen, 2013 [82]
	1. Gait patterns with/without GJH 2. Knee ROM with/without GJH	8–15	1. Differences with/without GJH 2. GJH > control	Fatoye, 2011 [49]
	Trunk and head stability in gait with/without GJH	9–11	GJH < control	Falkerslev, 2013 [47]
Pain and injuries	1. Chronic pain 2. Dislocation/subluxation	10	1. Not associated 2. GJH > control	Remvig, 2011 [94]
	Musculoskeletal pain	6–17	GJH > control	Abujam, 2014 [12]
	1. Pain area counts 2. Pain lasting more than 3 months 3. Pain worsened by sports	14	1. GJH > control 2. Not associated 3. Not associated	Morris, 2017 [76]
	Musculoskeletal pain	Av. 13.9	GJH > control (worse pain)	Tobias, 2013 [109]
	1.Musculoskeletal pain 2. Injuries	8	1. Not associated 2. Not associated	Jull-Kristensen, 2009 [66]
	1. Musculoskeletal problems 2. Pain and discomfort	18–24	1. GJH > control 2. GJH > control	Akkaya, 2022 [14]
	1. Pain occurrence 2. Pain severity	10–18	1. GJH > control 2. Not associated	Rejeb, 2019 [93]
Proprioception	Knee proprioception	8–15	EDS < control	Fatoye, 2009 [48]
	Knee proprioception	8–16	Not associated	Pacey, 2014 [87]
	Foot proprioception	5–14	GJH < control	Akkaya, 2023 [15]

(3) Cluster 3: Other physical implications of GJH				
Physical condition	Disorder	Age	Association with GJH	References
Gastrointestinal problems	1. Functional 2. Abdominal pain IBS	7–12	1. Not associated 2. Not associated	Shulman, 2020 [104]
	1. Functional constipation 2. Functional dyspepsia 3. Functional abdominal pain 4. Functional defecation disorders 5. Functional nausea and vomiting disorders	Av. 12.3	1. Not associated 2. Not associated 3. Not associated 4. Not associated 5. GJH > control	Ortiz-Rivera, 2022 [86]
	Presence of GJH and fibromyalgia in children with gastrointestinal disorders	10–18	32.5% GJH 64.2% fibromyalgia	Şahin, 2023 [96]
Autonomic system	1. OH 2. POTS 3. Syncope	5–24	1. Not associated 2. Not associated 3. Not associated	Chelimsky, 2016 [29]
	1. OH 2. POTS	10–18	1. Not associated 2. Not associated	Velasco-Benitez, 2020 [112]
	1. OH 2. POTS	10–18	1. Not associated 2. Not associated	Velasco-Benitez, 2021 [113]
	POTS	14–17	3. GJH > control	Boris, 2021 [24]
Central sensitization	1. Hyperalgesia, low pain threshold 2. CS 3. FD	12–19	1. GJH > control 2. GJH > control 3. GJH > control	Bettini, 2016 [20]
	1. CS, pain sensitivity 2. FD	Av. 15.75	1. GJH > control 2. GJH > control	Bettini, 2018 [19]
Reproductive system	Sex hormone binding globin	10–18	Higher SHBG linked to more hypermobile joints	Graf, 2019 [55]
	Menstrual problems in women with GJH*	11–18	80% showing heavy/very heavy bleeding	Kendel, 2019 [69]
Urinary and voiding dysfunction	1. Daytime and nighttime urinary incontinence 2.Constipation 3. Fecal soiling	5–12	1. Girls with GJH > control girl 2. Trend toward girl with GJH > control girls 3. Trend toward boys with GJH > control boys	De Kort, 2003 [38]
	1. Urinary tract infection 2. Constipation	5–14	1. Girls with GJH > control girls 2. Boys with GJH > control boys	Kajbafzadeh, 2014 [67]
	Vesicoureteric reflux*	≥ 6	61.484% of patients had GJH	Tokhmafshan, 2020 [110]
Bleeding symptoms	Bleeding symptoms	6–21	75% of participants had an abnormal bleeding score	Kendel, 2022 [70]
	1. Bleeding symptoms in children with EDS 2. GJH in children with hematosis	9–18	1. 56% of children with EDS had abnormal bleeding symptoms 2. 28% of children with hematosis had GJH	Hickey, 2016 [58]
Eye care	Anterior segment analysis and corneal biomechanical properties	Av. 10.98	Not associated	Bayramoğlu, 2020 [18]
Dental status	1. Plaque 2. Tooth bleeding 3.Tooth mobility 4. Decay, filled and missing teeth	6–16	1. GJH > controls 2. GJH > controls 3. Not associated 4. Not associated	Demir, 2021 [39]
Chronic illness	Fibromyalgia	11–18	Not associated	Barçak, 2015 [16]
	CFS	≥ 10	CFS patients had more GJH than control (60% versus 24%)	Barron, 2002 [17]
*No control group				

(4) Cluster 4: Psychosocial implications of GJH				
Subject of interest	Measured	Age	Association with GJH	References
Mental health disorders	GJH in children with/without anxiety	5–17	More GJH in anxiety versus without	Bulbena-Cabre, 2019 [26]
	GJH in children with/without anxiety	8–15	More GJH in anxiety versus without	Parvaneh, 2020 [91]
	Anxiety disorders in children with/without GJH	9	GJH > control	Ezpeleta, 2018 [45]
	Eating disorders in students with/without GJH	18–23	Correlation between disordered eating and BS	Can, 2022 [27]
Quality of life	1. Quality of life perception 2. Pain intensity	8–15	1. GJH < control 2. GJH > control	Fatoye, 2012 [50]
	1. Functional impairment 2. Quality of life 3. Fatigue 4. Illness perception	12–20	1. GJH/EDS > control 2. GJH/EDS < control 3. GJH/EDS > control 4. GJH/EDS > control	Mu, 2019 [78]
	Fatigue, pain and stress incontinence*	6–16	Negatively correlated with quality of life in children with GJH	Pacey, 2015b [90]
	Readiness to self-manage symptoms, depression, functional disability and challenges regarding EDS*	8–18	Readiness to self-manage symptoms positively correlated with familial support and longer time since diagnosis, pain and physical symptoms negatively correlated with quality of life in children with EDS	Bieniak, 2022 [21]
	Functional disability, mental health and social support	8–18	Functional disability was related to more mental health disorders regardless of social support	Bieniak, 2022b [22]
	Pain, number of physical symptoms, fatigue and mental health disorder	8–18	Correlated with FD in children with GJH	Tran, 2020 [111]
	1. Functional performance 2. Quality of life	14	1. Not associated 2. Not associated	Schmidt, 2017 [101]
Learning disabilities	GJH in children with learning disabilities	6–7	Not associated	Davidovitch, 1994 [36]
	ADHD and ASD in children with symptomatic GJH*	6–18	16% were diagnosed with ADHD and 7% were suspected to have ADHD, 6.5% had ASD	Kindgren, 2021 [71]
*No control group				

(5) Cluster 5: Treatments				
Type of treatment	Procedure description	Age	Improvement in:	References
Intervention	1. Education 2. Body consciousness and stability training	12–13	1. Pain 2. Pain due to physical activity 3. Injury	Gyldenkerne, 2017 [56]
	Physiotherapy and exercises	5–17	GJH symptoms	Birt, 2014 [23]
	Ride therapy	7–14	1. Knee stability 2. Muscle strength 3. Proprioception	Mosulishvili, 2013 [77]
	1. Physiotherapy 2. Psychological counselling 3. Occupational therapy	9–18	1. Pain 2. Depression and anxiety 3. Social functioning 4. Physical functioning	Revivo, 2019 [95]
	Knee exercises in full ROM versus knee exercises in neutral ROM	7–16	1. Knee pain 2. Thigh strength 3. Physical health 4. Mental health	Pacey, 2013 [88]
	Transcutaneous electrical nerve stimulation	5–14	Incontinence symptoms	Salem, 2010 [97]
Rehabilitation	Strengthening and stability training	16	Chest pain	Nash, 2017 [79]
	Strengthening and stability training	10	Elbow stability, strength and function	Karademir, 2022 [68]
Orthopedic aids	Neoprene Wrist/hand splints	14	None	Frohlich, 2012 [53]
	Foot orthotics	5–15	1. Gait synchrony 2. Step homogeneity	McDermott, 2018 [74]

(6) Cluster 6: Aesthetic sports context				
Type of sports	Measurements	Age	Effects of participation	References
Ballet	1. Spine curvature and mobility 2. Spinal kyphosis and lordosis	10	1. Ballet > control 2. Ballet < control	Nilsson, 1993 [83]
Dance	1. Pain and fatigue 2. School and cognitive functioning 3. Sleep and rest	6–16	1. Dance < control 2. Dance > control 3. Dance > control	Nicholson, 2017 [80]
Ballet and TeamGym	Quality of life	14	Lowest quality of life in ballet and TeamGym in comparison to other sports	Schmidt, 2017 [101]

The prevalence of GJH in different ethnic groups ranged between 9.4% (with a BS cut-off of ≥ 4) [56] and 36% (with a Carter–Wilkinson criteria cut-off of > 5) [52]. Most studies found higher prevalence of GJH in younger children [51, 52, 105, 118]. Findings regarding gender differences were inconsistent. Whereas most studies found GJH was more prevalent in girls [16, 30, 54, 56, 61, 103, 119], in others it was just a trend [41, 52, 99, 105, 117]. One study found a higher prevalence in boys [118] and one study found a higher prevalence in boys aged 6–10, while prevalence was higher in girls aged 11–15 and overall [108], and two studies found no gender differences [25, 94].

Studies also investigated the prevalence of GJH in association with musculoskeletal conditions such as scoliosis or pain, showing no links with scoliosis [25, 40, 56, 94]. One study observing GJH and fibromyalgia found no correlation between them, with only one child meeting criteria for both conditions [16]. A different study looked at GJH’s prevalence in children with gastrointestinal problems, finding no association between these conditions [104]. Another study examined the prevalence of GJH in patients with vesicoureteric reflux and found that 66.7% of boys and 57.7% of girls had GJH, indicating links between these conditions as its prevalence in this cohort was much higher than in the general population [110]. Two studies looked at GJH’s prevalence in Postural Orthostatic Tachycardia Syndrome (POTS), with one study finding correlations between symptomatic GJH and POTS and the other finding no such correlation with GJH [24, 113]. Another study observed the prevalence of GJH in children with anxiety [91] and found it was higher in comparison to children without anxiety (see Table 3(1:B)).

One study in this cluster looked at Body Mass Index (BMI) and GJH and found they were negatively correlated, so that underweight children had a higher percentage of GJH [98]. Another study examining GJH’s importance in pre-pubertal children found more joint pain in children with GJH, which correlated with parents’ musculoskeletal problems, and higher frequency of flat feet than in healthy comparisons [116]. Two studies looked at general signs and symptoms of GJH and classified children into subcategories with possible different complications and trajectories. The first ran a multifactorial analysis, identifying five distinct GJH clusters [89]. The second study was longitudinal, following children for 3 years. It identified three subgroups according to symptom severity [100]. Functional impairment at baseline was predictive of reduced walking distance and decreased quality of life. Four underlying constructs contributed to disability: multi-systemic affects, pain, fatigue, and loss of postural control.

The final type of studies in this cluster assessed screening tools for GJH. Out of the six articles included in this review, five used the BS. The earliest study examined BS’s validity in children, deeming it valid [106]. The second study looked at inter-test reliability of two different BS versions and found moderate to substantial reproducibility for both methods, when following standardized protocols [63]. Another study compared the BS with the Hospital del Mar criteria, and found more children had GJH using the latter than a cut-off of ≥ 4 on the former with a prevalence of 34% versus 12%, respectively [84]. The fourth study examined BS’s suitability for children with intellectual disability. Agreement between judges was moderate and inter-class correlations yielded excellent reliability. GJH’s prevalence in this population was similar to children without intellectual disabilities (8%), with correlations between GJH and age and gender, suggesting its usage was feasible and reliable in this cohort [92]. The fifth study looked at reproducibility of GJH assessment online versus in-person, showing that while more children were classified as having GJH in the in-person mode, agreement on BS score was fair to excellent. Tools measuring upper limb and lower limb hypermobility yielded much poorer agreements [59]. The final study on screening methods investigated whether functional tests of the pelvic-hip complex and trunk flexibility could screen for GJH. There was no difference in functional tests between children with and without GJH (as measured by the BS), indicating this was not a valid tool for screening GJH [31].

#### Orthopedic implications of GJH

The orthopedic cluster is the largest cluster in this review, covering topics such as certain joints and their biomechanics, gait patterns, joint pain, and injuries. Joints of interest included the spine and scoliosis, knees, hips, temporomandibular joint dysfunction, and clicking and more (see Table 3(2:A)).

Studies in this cluster also examined various orthopedic implications. Findings regarding motor competence in children and adolescents were inconsistent. Two studies showed a decrease in muscle strength, with an impairment in exercise capacity [14, 57]. A study looking at GJH in typically developing children and children with motor developmental impairments revealed some differences in GJH between the groups. However, GJH failed to explain differences in variance beyond neuromuscular performance, indicating no association between motor performance development and GJH [115]. Two studies showed the reversed link, where children with GJH performed better than their healthy counterparts. One of the studies reported girls with GJH performed better in vertical height jump [94], with the other showing children with GJH had better dynamic balance and shorter reaction times than those without GJH [66].

The two studies looking at developmental coordination disorders (DCD) found no difference in DCD among children with and without GJH [75], with a tendency of children with GJH to score higher in dexterity than children without [37].

Studies examining gait in youth with GJH showed gait pattern differences between them and healthy controls. One study found differences in kinetics, lower peak joint moments, and smaller step width in children with GJH [82]. A second study showed knee movement patterns significantly differed in children with and without GJH [49]. A third paper found that children with GJH had decreased lateral trunk stability accompanied by decreased head stability while walking compared to controls [47].

Studies looking at arthralgia, musculoskeletal pain, and injuries in GJH found some associations between them. The first study reported 17.6% of participants had Ehlers-Danlos Syndrome (EDS) [94]. Several studies found a significantly higher occurrence of musculoskeletal pain and arthralgia in children with GJH compared to those without and one study found that children with EDS had higher pain intensity, more discomfort and worse life quality than controls [12, 14, 25, 50, 76]. In contrast, one study found GJH was only related to worse pain in children who reported any pain [109], while two studies found no association between GJH and widespread pain [66, 94]. Most common musculoskeletal complaints in children with GJH were ankle sprains (31.3%), exercise-related pain (15%), arthralgia (12.6%), and back pain (10.6%) [12, 25]. Regarding injuries, results were conflicting, with one study finding higher prevalence of dislocations/subluxations in children with GJH compared to controls, another study finding a higher BS was associated with an increased risk of injury, but not with injury severity, while a third study did not find more injuries in the GJH group [66, 93, 94] (see Table 3(2:B)). Two follow-up studies found that while GJH in childhood increased the risk for developing musculoskeletal pain in adolescence, it did not affect physical functioning, daily activities or participation in them [107, 109]. Three studies examined proprioception in GJH, showing conflicting results. While two studies found reduced proprioception in children with GJH [15, 48], another study found no differences regarding proprioceptive acuity between children with and without GJH [48, 87].

#### Other physical implications

While GJH’s most common implications are joint and musculoskeletal system involvement, it has other physical implications. Three studies examined gastrointestinal problems. Whereas the first study found children with functional nausea and vomiting disorders had more GJH [86], the second study showed no correlation between GJH and gastrointestinal involvement [104]. A third study looked at the interlinks between GJH and fibromyalgia in children with gastrointestinal disorders and suggested all three conditions were interlinked, perhaps through emotional distress, as presence of GJH and fibromyalgia in this cohort was rather high (32.5% and 64.2%, respectively) [96].

Three studies found no association between the autonomic system and GJH, with no links between GJH and orthostatic hypotension, tolerance, and POTS, and a trend toward more dizziness in the GJH group [29, 112, 113], while one study showed that among children with POTS, 61.7% had symptomatic GJH, indicating a link between these conditions [24]. Some links were found between central sensitization and GJH. One study showed GJH was associated with more hyperalgesia and lower pain threshold. BS scores were also correlated with central sensitization and functional disability [20]. A second study found that although adolescents with GJH had higher comorbidity with chronic pain and functional disability, this was only true for a subjective pain level assessment [19]. Two studies explored GJH’s relation to the reproductive system. The first observed hormone levels, finding that higher levels of sex‐hormone‐binding globulin serum in both genders were associated with a greater number of hypermobile joints [55]. The other study focused on heavy menstrual bleeding, finding that 80% of young women reported heavy menstrual bleeding, 70% reported menses interfering with work and social life, and 87% reported limitations in physical activity [69].

Three studies examining urinary problems, voiding dysfunction and vesicoureteric reflux found a significant bi-directional association with GJH [38, 67, 110]. Patterns for boys and girls were different—boys showed more constipation problems, whereas girls showed more incontinence and urinary tract infections[38].

Two studies that observed bleeding symptoms in relation to GJH showed that patients had much higher abnormal bleeding symptoms than their prevalence in the general population [58, 70], with the most common bleeding in the cohort being oral bleeding (74.1%), easy bruising (59.3%), and bleeding with minor wounds (42.0%) [70]. It was also found that 28% of hemostasis patients had GJH and 15.6% had EDS [58].

One study looking at eye care in children with GJH found no significant differences between children with and without GJH regarding biomechanical and topographic parameters and no increased risk of keratoconus in children with GJH [18].

One study looking at dental status in children with GJH found higher prevalence of plaque and tooth bleeding in the GJH group compared to control group, while there was no difference in decay, tooth filling, and missing teeth, as well as in tooth mobility [39].

Three studies examined the link between GJH and chronic illness. Two of which looked at fibromyalgia, with one showing no association between the two conditions, while the other suggested they might have underlying pathways [16, 96]. A study looking at children with and without Chronic Fatigue Syndrome (CFS) found significantly higher BS scores in CFS patients [17] (see Table 3(3)).

Three papers compared three groups: children with symptomatic GJH; children with asymptomatic GJH; and healthy children. Children with symptomatic GJH had significantly higher total ROM, skin extensibility and lower bone density, lower diastolic blood pressure, and higher degradation products in urine compared to children with asymptomatic GJH. When compared with healthy controls, children with asymptomatic GJH had higher total ROM and more skin extensibility [42]. Decreased absolute peak and relative oxygen consumption were found in both patient groups, indicating lower exercise tolerance [43]. The third study found higher pain intensity in children with symptomatic compared to asymptomatic GJH. Differences also emerged in balance and activity and children with symptomatic GJH required more rest than healthy children [102].

#### Psychosocial implications

The psychosocial implications of GJH on children and adolescents’ cluster included studies with three different foci: (1) mental health disorders; (2) life quality and functioning; (3) neurodevelopmental disorders. Three papers studied Anxiety Disorders (AD), finding a significant bi-directional correlation with GJH [26, 91]. The most prevalent ADs in this cohort were separation anxiety, social phobia, and fears of physical injury [45]. One study looked at Eating Disorders (ED) and found GJH had a weak significant link with reported disordered eating [27]. Seven studies focused on quality of life and found it was lower in youth with GJH in comparison to healthy controls. Pain along with number of symptoms, fatigue, and stress incontinence predicted quality of life, dysfunction, anxiety, and depression in the GJH group [21, 22, 50, 78, 90]. Children and adolescents with EDS reported their greatest challenges were managing their physical symptoms, not being able to do things their peers do and feeling left out [21, 111]. Social support did not mitigate the negative impact EDS had on quality of life or mental health [22]. Only one study showed no association between GJH and quality of life [101]. Two studies explored neurodevelopmental disorders in children and adolescents with GJH. Whereas one found GJH was not associated with learning disabilities [36], a newer study found that both Attention Deficit and Hyperactivity Disorder (ADHD) and Autistic Spectrum Disorders (ASD) were higher in the GJH group than in the general population [71].

#### Treatments

This cluster included three types of treatments: interventions, rehabilitation, and orthopedic aids for children with GJH. Five studies offered patients physical interventions, which included either physiotherapy, strengthening, and movement training or a combination, often accompanied by education about GJH. All studies found improvement in pain, injury, and/or strength post-intervention [23, 56, 79]. One study compared knee strengthening exercises either in full ROM or only in a non-hyperextended ROM for children with GJH. It showed both groups had significant improvements post compared to pre-interventions; however, overall, working at a hyperextended range was more beneficial, especially for self-esteem, behavior, and mental health [88]. One study showed that ride therapy was favorable to therapeutic gymnastics in increasing muscle strength, proprioception, and knee stability [77]. A study conducting an interdisciplinary intervention, combining physical therapy, psychological counseling, and occupational therapy, showed improvements in pain, physical functioning, and mental health [95] (See Table 3(5)). Another study treated children with hypermobility and an overactive bladder using transcutaneous electrical nerve stimulation. It found 77% of the children had complete to mild improvement in incontinence symptoms, while only 23% showed no improvement [97].

Two studies were case studies looking to rehabilitate children with a specific complaint and underlying GJH, both offered a strengthening and stability training program and reported patients’ full recovery [68, 79].

Two studies explored orthopedic aids for treating GJH’s implications. A paper looking at Neoprene wrist/hand splints found they were not beneficial in reducing pain or improving children with GJH’s handwriting [53]. However, a study examining effects of foot orthotics on gait patterns in children with GJH demonstrated improvements in gait synchrony, and less step variability [74].

#### Aesthetic sports

This cluster included only three studies. The first study observed differences in spine curvatures of ballet students in comparison to age-matched non-dancers. It found significant differences between the groups for all spine mobility variables except total ROM in the lumbar spine. Moreover, kyphosis and lordosis were less prominent in ballet dancers [83]. Another study examining difference between dancers and non-dancers with GJH found dancers reported less pain, fewer pain-related problems, and less body areas affected by pain. Furthermore, dancers perceived school functioning, sleep/rest, cognitive, and total fatigue levels as better [80]. A study looking at GJH in elite athletes found the highest prevalence of GJH in ballet and TeamGym. These disciplines also showed the lowest life quality. There was no difference between athletes with and without GJH in neither life quality, muscle strength nor in injuries [101].

### Mapping changes in the field

**Fig. 2:**
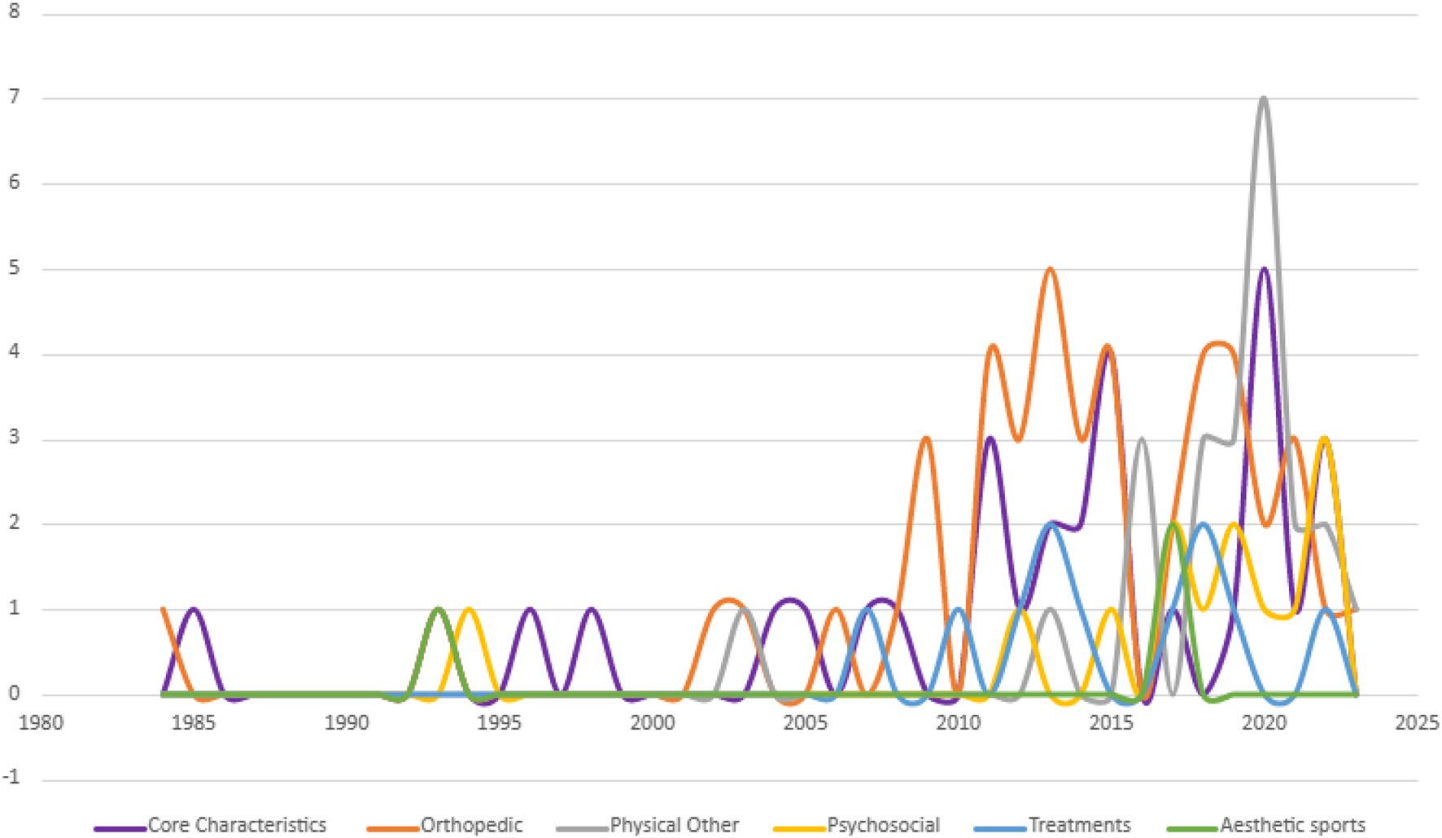
Number of studies by year of publication

This review identified changes that occurred in the research field throughout the years. First, there has been growth in the volume of GJH studies in children and adolescents in recent years. While research began in the 1980s, publications were sporadic until the second decade of the twenty-first century. Frequency increased since 2011, with 2020 being publications’ peak year (see Fig. 2).

Second, a change in the studies’ foci was identified. Early papers mostly belonged to either the GJH core characteristics or the orthopedic cluster. Most studies in the physical cluster came out in the second decade of the twenty-first century. Some newer studies incorporate a biopsychosocial approach, examining quality of life, with the first study published in 2011 and the first mental health disorders associated with GJH study published in 2018 (see Fig. 2).

## Discussion

This review aimed to synthesize and evaluate evidence of the prevalence of GJH among children, adolescents, and young adults, and how it is measured. The review also examined evidence of GJH’s physical and psychosocial implications in this cohort, along with GJH in a context of aesthetic sports. The review included 107 studies that fell into 6 thematic clusters, with a large body of research regarding core characteristics of GJH and its physical and especially musculoskeletal implications. Very few studies, all published in the last decade, examined GJH’s psychosocial implications, with even fewer looking at GJH in aesthetic sports. Another type of studies identified and included was treatments for GJH’s symptoms. The review also identified changes and trends in the field over time.

### Prevalence

The prevalence of GJH in different studies ranged between 9 and 36% [52, 56], similar to reports in the general population (2–57%) [4]. Prevalence differences are explained by age, gender, ethnicity, activity, and measurement. Indeed, this review found age was negatively correlated with GJH, with less GJH in older children [41, 52]. Most studies included in the review found that girls had higher prevalence of GJH, while some failed to show gender differences. This is possibly due to papers examining pre-pubertal children who show no such differences. This is supported by Forelo et al.’s study [52] that found no gender differences in younger children, with significant differences in adolescents.

In terms of measuring GJH, studies employing the BS deemed it a suitable tool for children, including those with intellectual disabilities, when following a standardized protocol and a cut-off of ≥ 5 [63, 92, 106]. However, even within the current review, prevalence studies suggested cut-off points, ranging between 4 and 7. Recent papers on the BS argue that it lacks standardization, has an unclear administration procedure, and measures joints not necessarily indicative of GJH [7]. Hence, more studies are warranted to investigate BS’s validity.

While some older studies used tools such as the Carter–Wilkinson criteria, almost all studies conducted in the twenty-first century used the BS. In several studies, participants were also tested for EDS; however, unlike GJH’s screening, EDS must be diagnosed by a clinician, making it harder to utilize in multidisciplinary research. In practice, some other physical markers are used (e.g., skin markers, anthropometrics); however, this review found no evidence of their usage in research.

### Physical implications

The understanding of GJH as a condition affecting not only the joints, but other areas of the body is rather new. This might explain why more recent studies included in this review looked at other physical implications of GJH. While some of GJH’s accompanying symptoms are local, others are global and systematic with studies exploring both types of manifestations [8]. Findings regarding the gastrointestinal and autonomic systems were in contrast to well-established links in adults, as several studies in this review found no differences in youth with GJH [120]. One possible explanation is that the studies did not differentiate between symptomatic and asymptomatic GJH, this is supported by the single study that looked at symptomatic GJH in children with POTS and found it was highly prevalent in this cohort [24]. However, it is unlikely, as other differences such as higher somatization and depression were observed in the studies that failed to find links between GJH and autonomic and gastrointestinal system involvement [104]. Authors suggested these findings could indicate real differences between adults and children, or limitations of the studies due to tools that lack sensitivity, or selection bias [112]. More research is needed to resolve these discrepancies.

Compatible with research on adults [3], central sensitization was linked with GJH, with higher hyperalgesia and a lower pain threshold in children and adolescents [19, 20].

Studies looking at gynecologic implication showed most females with GJH reported heavy menstrual bleeding [69]. These finding are consistent with gynecological problems in adult women [121]. Urinary and voiding dysfunction, which showed links with GJH in this review, have shown similar associations in adults and especially in woman, who suffer from urinary tract dysfunction, bladder problems, and prolapses [122].

### Psychosocial implications

Studies observing psychosocial aspects of GJH are becoming more common and suggest such links might involve pathophysiological pathways [123]. Compared to the volume of studies examining GJH’s psychosocial implication in adults, those are still scarce in young people. Most psychosocial studies in this review focused on quality of life, with only four looking at mental health disorders. Furthermore, only anxiety and eating behaviors were examined in this cohort, while adult studies have tackled many more mental health disorders [2].

Studies on anxiety found GJH and anxiety had a similar bi-directional link to that seen in adults with a higher prevalence of GJH in people with anxiety, as well as a higher prevalence of anxiety in people with GJH than in those without [2, 26, 91, 123]. However, specific ADs in adults with GJH are different from those found in children [124], sharing only social phobia in both populations [45]. Separation anxiety might be linked with GJH only in children, as it mostly manifests in childhood. It might stem from parents of children with GJH seeing them as weaker and requiring more care. This parenting style could, in turn, lead to separation anxiety. Fears of injury could be explained due to children with GJH experiencing more pain, injuries, and medical encounters. However, more comprehensive studies are needed to better understand the mental health of children with GJH.

The study looking at EDs found a positive link between reported disordered eating and BS scores [27]. However, only four participants in the study met the criteria for an ED. Previous studies did show links between GJH and EDs [125].

Studies on quality of life in youth with GJH found a significant decrease in their life quality and increased dysfunction. These were linked to pain, anxiety, incontinence, and fatigue. Findings in adults with GJH also showed decreased life quality and disability [126]. Further research is required to determine the full extent of the impairment and hardships this cohort experiences.

In the current review, only two papers looked at learning difficulties in children with GJH, with conflicting findings. While one study reported no such link [36], the second found both ADHD and ASD were associated with GJH. Previous studies found links between ADHD, ASD, and GJH, and suggested common pathways, including DCD, fatigue, and sensory anomalies [125, 127].

### Interventions

All treatments included in this review showed improvements post-intervention for youth with GJH compared to pre-intervention, apart from a study about wrist/hand splints. Physiotherapy, strengthening and stability exercises, psychological counseling, occupational therapy, and ride therapy were able to decrease pain, improve strength, life quality and specific orthopedic problems. Only a few studies tackled interventions for people with symptomatic GJH, with inconclusive results [128]. The existing literature recommends encompassing interventions, targeting both physical and psychosocial aspects [8]. However, most interventions are local, focusing on symptoms, instead of targeting the root of the problem. More research on this topic is needed.

### Aesthetic sports

Studies on dancers with GJH reported fewer back problems, better school functioning, better sleep/rest, and less fatigue and pain in comparison to non-dancers with GJH [80, 83]. Studies comparing well-being and functioning of dancer and non-dancer adults with GJH, found that while dancers had higher muscle strength and performed better on functional tests, they reported more fatigue and pain than non-dancers [129]. Pain and fatigue might increase in those with GJH later in life, accounting for such discrepancies. A third study found that within a group of elite athletes, dancers and gymnasts had the highest prevalence of GJH, which is consistent with findings in collegiate dancers [129]. Dancers and gymnast also reported the lowest quality of life, a finding compatible with results in adults [101]. Studies regarding aesthetic sports/dance are scarce across all populations and the interlinks between them and GJH should be further investigated.

### Trends in the literature

This review revealed trends in research—notably, the volume of studies on childhood and adolescence GJH has increased in the last decade. Perhaps due to the growing understanding of the implications and trajectory of GJH, alongside the understanding that early interventions could help prevent its symptoms from worsening [3]. So far, 2020 has been the peak year for number of publications, closely followed by 2022, indicating a growing interest in the research field.

The recent increase in the studies’ volume does not necessarily reflect their quality. Most studies included in this review were cross-sectional. Whereas some studies were more comprehensive, recruiting large samples, allowing follow-ups and performing clinical exams, those were rare. Some newer studies still lack rigorous and robust methods, with many recruiting small, convenience samples, and only screening for GJH using the BS. Another problem that arose was the lack of coherent terminology. Although 2017 accelerated a shift toward new taxonomy, not all researchers have adopted it, creating confusion around terms referring to symptomatic GJH.

Changes were also observed in the focus of the research field throughout the years. From focusing on prevalence and musculoskeletal problems, the range expanded to include other physical implications (e.g., gastrointestinal and cardiovascular involvement), and a psychosocial perspective, looking at life quality and mental health. It can be argued these changes reflect the growing understanding regarding GJH in the general population as a global condition, affecting the entire body as well as the mind [123]. Moreover, it is likely to reflect a shift in the medical field to the biopsychosocial model, looking at aspects of patients’ lives other than biological ones [130].

## Limitations

This review’s limitations arise from the large number of studies included, which restricted examining the implications of GJH in detail, while only outlining a wider understanding of it in children and adolescents. Moreover, studies included were mostly of low methodological quality; hence, results should be observed with caution. Some topics in this review included only one or two studies, so it is harder to draw conclusions on them. Furthermore, this study did not include HCTDs other than GJH and EDS, which limited its scope.

## Conclusion

The current review included 107 studies exploring GJH in children, adolescents, and young adults. It examined GJH’s prevalence in this population, tools used to measure it, its physical along with psychosocial implications, treatments to improve its debilitating effects and GJH in an aesthetic sports context. The review found a growing interest in GJH in this cohort, especially regarding non-musculoskeletal physical implications and psychosocial aspects. Prevalence varied between different ethnic groups and as a function of age, gender, and measurement. BS was the most widespread tool to measure GJH. While links with many physical conditions emerged, these seem fewer than in an adult population. Psychosocial implications and decreased quality of life resembled findings in adults. The proposed interventions helped ease many impeding symptoms; however, further research is warranted to determine the full scope of GJH’s impairment in childhood, especially regarding the psychosocial aspects, as well as effective treatments.

## Data Availability

The authors confirm that the data supporting the findings of this study are available within the article [and/or] its supplementary materials.
